# The Anatomy of a Weight Recidivism and Revision Bariatric Surgical Clinic

**DOI:** 10.1155/2014/721095

**Published:** 2014-02-11

**Authors:** C. J. de Gara, S. Karmali

**Affiliations:** ^1^University of Alberta, 2-590 Edmonton Clinic Health Academy, Edmonton, AB, Canada T6G 1C9; ^2^Bariatric Revision Surgery Clinic, Alberta Health Services, Edmonton, AB, Canada T5H 3V9; ^3^Surgical Director Weight Wise Bariatric Clinic, Minimally Invasive Gastrointestinal and Bariatric Surgery, Alberta Health Services, Edmonton, AB, Canada T5H 3V9

## Abstract

*Abstract*. Weight recidivism in bariatric surgery failure is multifactorial. It ranges from inappropriate patient selection for primary surgery to technical/anatomic issues related to the original surgery. Most bariatric surgeons and centers focus on primary bariatric surgery while weight recidivism and its complications are very much secondary concerns. *Methods*. We report on our initial experience having established a dedicated weight recidivism and revisional bariatric surgery clinic. A single surgeon, dedicated nursing, dieticians, and psychologist developed care maps, goals of care, nonsurgical candidate rules, and discharge planning strategies. *Results*. A single year audit (2012) of clinical activity revealed 137 patients, with a mean age 49 ± 10.1 years (6 years older on average than in our primary clinic), 75% of whom were women with BMI 47 ± 11.5. Over three quarters had undergone a vertical band gastroplasty while 15% had had a laparoscopic adjustable gastric band. Only 27% of those attending clinic required further surgery. As for primary surgery, the role of the obesity expert clinical psychologist was a key component to achieving successful revision outcomes. *Conclusion*. With an exponential rise in obesity and a concomitant major increase in bariatric surgery, an inevitable increase in revisional surgery is becoming a reality. Anticipating this increase in activity, Alberta Health Services, Alberta, Canada, has established a unique and dedicated clinic whose early results are promising.

## 1. Introduction

Inevitably some surgical procedures will fail. This is as true for obesity surgery as it is for many areas of health care. Examples are many within the gastrointestinal tract, such as highly selective vagotomy for peptic ulcer disease [1], the Angelchik procedure for reflux [2], and of course vertical band gastroplasty [3]. Even procedures that have an ongoing place in the surgical armamentarium such as Nissen Fundoplication [4] have changed their once high frequency rate to very much more restrictive indications. This occurred as the growing weight of evidence emerged as to a procedure's ultimate effectiveness or otherwise [5].

Weight regain remains the scourge of any and all obesity management strategies. With only 6% of nearly 1,000 subjects maintaining a 5% weight lost after 6 years [6] and a third of weight loss being regained within a year of non surgical weight lost interventions [7].

As with primary obesity, weight recidivism is multifactorial. These factors have been neatly categorized by Dr. Arya Sharma into mental (mood, anxiety, ADD, sleep, personality, addiction, etc.), mechanical (osteoarthritis, pain, GERD, and sleep apnea), monetary (education, employment, income, and insurance), and Metabolic (type II diabetes, dyslipidemia, hypertension, cancer, and infertility) disorders (http://www.drsharma.ca/the-4ms-of-obesity-assessment-and-management.html). To this list the postbariatric surgical failure due to mechanical problems can be added. The burgeoning demand and inevitable entrepreneurial opportunities that obesity surgery provides have further fuelled the weight recidivism problem. A rush to offer bariatric surgery without adequately addressing the multitude of factors listed above will lead to a significant management failure rate [8]. Not all patients should be offered bariatric surgery merely because they are obese. Similarly, not all patients with weight recidivism should be offered revisional surgery. The problem of managing weight recidivism is further compounded by poor long term follow-up. It is well acknowledged that obesity is a life long struggle, yet few intervention studies extend much beyond 3–5 years [9].

Multidisciplinary clinics have a demonstrated effectiveness across a myriad of medical disorders, for example, pain, rheumatoid arthritis, pulmonary medicine, migraine clinics, and cancer clinics (http://scholar.google.ca/scholar?q=multidisciplinary+clinical+effectiveness&hl=en&as_sdt=0&as_vis=1&oi=scholarl&sa=X&ei=sIDEUFGSGKaaiAKP3IGoBQ&ved=0).

Within the obesity world multidisciplinary clinics are also demonstrating an increase in popularity and effectiveness, being part of both academic and nonacademic obesity programs [10].

## 2. Philosophy of Care

Within the weight recidivism and revision bariatric surgical clinic of Alberta Health Services a philosophy of care informs all decision making and patient management.

1. Patients managed in the clinic have failed previous bariatric surgery; they are symptomatic. Their symptoms range from weight regain and gastric obstruction to metabolic disturbances, through malnutrition.
2. Their care is grounded on an approach similar to that provided for the primary bariatric surgical patient within the provincial weight wise clinic, Alberta Health Services, in that surgery will inevitably fail if psychological, dietetic, and lifestyle issues are not concomitantly addressed.
3. Revisional surgery is complex; it frequently needs to be performed in an open laparotomy setting particularly when the original bariatric surgery was performed via a laparotomy incision. Laparoscopic strategies are becoming increasingly feasible especially for those whose primary surgery was done laparoscopically. Redo surgery has an inevitably higher complication rate than primary bariatric surgeries. These complications range from relatively minor wound infections to anastomotic failure, abscess formation, fistulae, sepsis, and even death.
4. Patients must not only be fully informed but must fully understand the risks, benefits, and the ultimate goals of care and demonstrate to the entire healthcare team a personal commitment and compliance, which will be lifelong.

## 3. The Multidisciplinary Clinic

**Figure 1:**
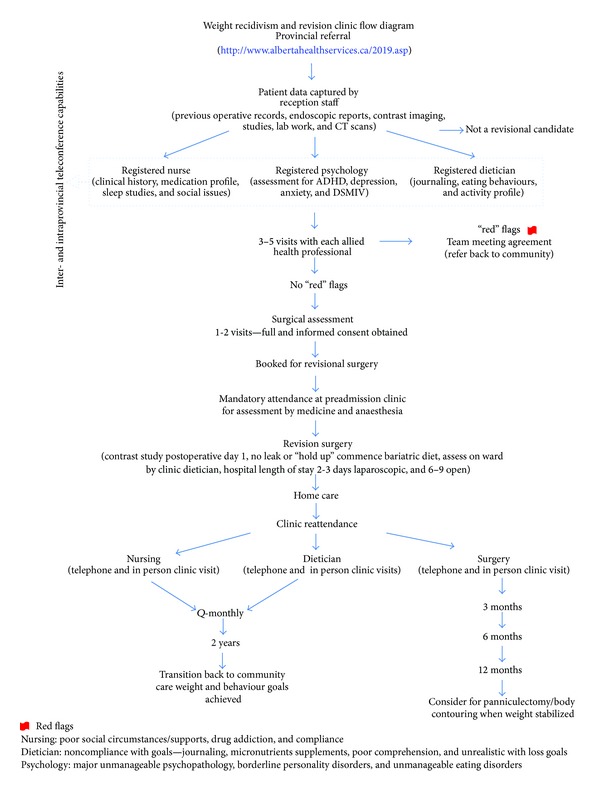
Multidisciplinary clinic flow diagram.

Compared with traditional office based practices, multidisciplinary clinics are intensive resource (in terms of both staff and time). Though often less efficient, there is a considerable weight of evidence as to their effectiveness in a multitude of disorders, particularly when these are complex and require the skills of allied health professionals above and beyond those that can be provided by physicians alone [11]. The weight recidivism and revisional bariatric surgical clinic in Edmonton, Alberta, is part of Alberta Health Services and the University of Alberta and has been modelled on the primary bariatric weight wise clinic of Alberta Health Services [12].  Figure 1 is a flow diagram of patient encounters through the weight recidivism revisional bariatric surgery clinic. A fundamental and key concept of these two clinics is that successful weight reduction for the obese patient requires multiple and thorough assessments by bariatric nursing, bariatric trained registered dieticians, and psychologists specifically trained in the mental health aspects of obesity. These services are further supported by internal medicine, gastroenterology, and psychiatry. Patient compliance and commitment to the program and the life-long struggle with obesity are demonstrated through diet journaling, attending up to 9 behaviour modification group therapy sessions, managing mental health issues and optimizing medical care for diabetes, hypertension, sleep apnoea, and so forth.

Table 1 Illustrates the differences between the primary weight wise clinic and the revision clinic. Recidivism patients are somewhat older than those in the primary clinic. The time spent in assessment and reassessment by nursing, dieticians, and psychologists is shorter. But particular attention is paid to understanding previous bariatric surgeries and defining the current anatomy through radiologic imaging and endoscopy. The decisions as to whether a particular patient is offered revision surgery are grounded in the concept of “RED FLAGS.”

## 4. Nursing Aspects

Following patient data acquisition by the administrative support staff, a pivotal point of contact for the patient is with the dedicated nursing staff. This registered nurse has had particular training and experience in assessing and managing the obese patient. Preliminary encounters are frequently by telephone. Social elements of the patient history as well as their general medical status are assessed.

The clinic has adopted a strategy of identifying “RED FLAGS” in which areas of potential concern are identified by each allied health professional. Being unresolved, these may lead to the patient being denied revisional surgery as, in the opinion of the multidisciplinary team, such a patient is likely to fail surgery.

For nursing, three major “RED FLAGS” are as follows.

1. The patient repeatedly asks “when am I going to meet the surgeon” tending to discount the importance and value of nursing encounter.
2. The patient insists that they know perfectly well how to eat healthily and that they are fully aware of a healthy diet and exercise programs and therefore do not need to be assessed by a dietician.
3. The patient is not interested in the process of life-style modification and they believe surgery is all that is necessary and they are not interested in complying with the additional strategies required by the program.

Nursing also uses this as an opportunity to perform the Epworth [13] sleep apnoea assessment tool and refer patient to formal sleep assessment and a CPAP machine as required.

## 5. Nutritional and Exercise Aspects

Critical in any obesity program is the role of the registered dietician and never more so in a weight recidivism revision clinic. The key to the success of such a program is the relationship that the obese patient must develop with their dietician. RED FLAGS that our dieticians have identified as major causes of concern are

1. noncompliance with goals: this would include noncompliance with maintaining a diet journal and noncompliance with taking vitamin and mineral supplements;
2. an unrealistic weight loses goal that remains unrealistic despite careful and repeated on-going education by the dietician;
3. mental health issues that make comprehension of the goals of care difficult, if not impossible, for the patient.

In addition to dietary guidance and recommendations, dieticians strongly reenforce the need for increased exercise, measured by the use of a pedometer and if necessary referral to an exercise specialist. The dieticians will also strongly reinforce the need for smoking sensation, with blood nicotine levels being measured to confirm adherence. On-going smoking is an absolute contraindication to revisional bariatric surgery given the high anastomotic ulcer rate [14].

## 6. Psychology Aspects

An important area that is increasingly being recognized as a vital component in obesity management is that of the psychological aspects of the disease [11]. It has been estimated that the upwards of 90% of obese patients suffer from either major or minor psychological problems. This compares with a figure of around 60% of the “normal” population (personal communication Dr. Brian Stonehocker, M.D., Assistant Clinical Professor, Psychiatry, University of Alberta). It is important to recognize that psychological or psychiatric issues are not themselves of cause for weight recidivism or contraindication to revision surgery. It is when these issues cannot be effectively managed or controlled that failure is more likely. From a psychologist's perspective RED FLAGS include

1. poorly controlled bipolar disorder or obsessive compulsive disorder;
2. severe depression and/or anxiety;
3. a history of multiple suicide attempts;
4. a borderline personality disorder;
5. multiple psychiatric hospitalizations, uncontrolled drug, and alcohol abuse [15].

A dedicated registered psychologist with considerable experience with the obese patient is a key member to the multidisciplinary revision clinic team.

## 7. Surgery

**Figure 2:**
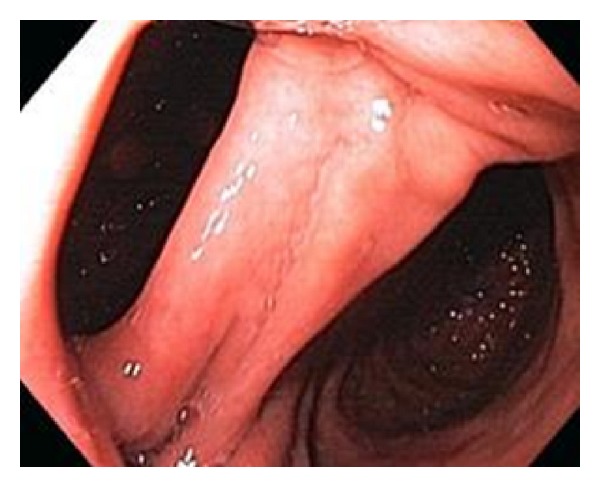
Gastrogastric fistula.

Once the patient has completed the rigorous dietary, psychological, and nursing assessment and reassessment, a surgical assessment will occur. This is an opportunity for the surgeon to review the previous bariatric surgical history as well as the current medical status of the patient. Contraindications to surgery through the “RED FLAG” process are reviewed. All patients are required to undergo an upper GI Endoscopy as well as a barium study of the oesophagus and stomach. These are used to gain a more complete understanding of current anatomy. Typically for the previous vertical band gastroplasty patient we would expect to see evidence of a gastrogastric fistula (Figure 2).

**Figure 3:**
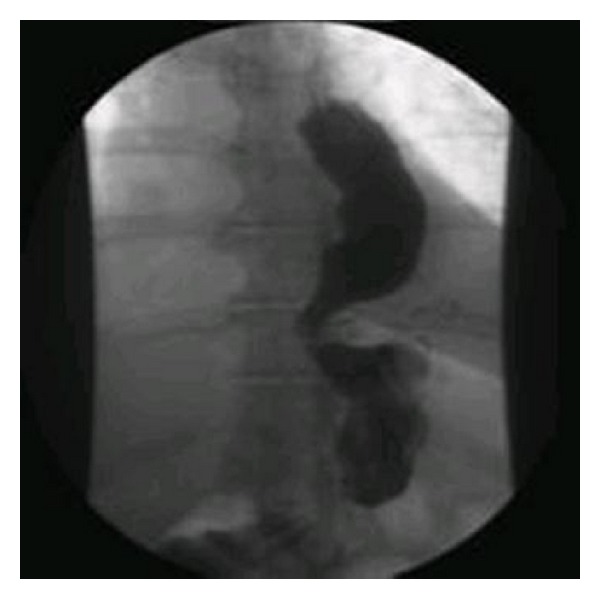
Pouch dilatation following sleeve gastrectomy.

This term is really a misnomer since a true fistula does not exist but rather there has been a restoration of normal gastric anatomy, with the vertical staple line disappearing over time, similar to that which occurs in the pylorus excluding procedure done for duodenal and pancreatic trauma [16]. Other anatomic variances that are examined for pouch dilatation in the previous gastric sleeve patient (see Figure 3) or the Roux-en-Y bypass patient.

A critical component in the surgical assessment is ensuring and confirming as to whether the patient and their social supports fully comprehend the risks and benefits of revision surgery. It is vital that a clear understanding that revisional surgery can be two or three times more demanding on both the patient and the surgeon as compared with the original primary procedure.

In addition to uncomplicated weight recidivism the multidisciplinary clinic is resourced to address additional problems that may be associated with failed primary bariatric surgery, for example, laparoscopic adjustable gastric band slippage and/or erosions, gastric outlet obstruction, malabsorption, dumping, and massive weight reduction.

Table 1 shows an audit of the recidivism clinic (*n* = 137) and comparison with the primary bariatric clinic (*n* = 863) revealing that these patients are on average age, at 49.8 ± 10.1 years versus 44.2 ± 11.5 years, having a similar male to female ratio (75% : 25%). Their initial bariatric procedure was 14 ± 8.1 years prior to clinic attendance and patients presented with an average BMI of 47 ± 11.5. Patients on average were seen 1.2 ± 8 times by nursing, 2.4 ± 1.9 times by dieticians, 2.6 ± 2.3 times by psychology, and further 0.9 ± 1.3 times by telephone or telehealth, representing significantly fewer visits than what occurs with the team compared with the primary clinic. Driven in large part by a symptomatic patient population, two-thirds of patients underwent surgery for weight recidivism alone, while the other third surgery for mechanical problems. These patients were on 5 ± 2.9 separate medications and had 4.3 ± 1.9 comorbidities in addition to their morbid obesity with 13% having type II diabetes, 25% sleep apnoea, 25% GERD, and 63% depression.

It is important to recognize that unlike most bariatric surgical clinics Alberta Health Services primary and revision clinic are very conservative with regard to offering surgery. This is evidenced by a 29% rate for primary patients and a 27% rate of recidivism and revision patients. The more common strategy of offering the majority of bariatric patients a surgical solution, then dealing with the failures in follow-up, is challenged by our approach to care. All previous vertical band gastroplasty patients were converted to a stapled Roux-en-Y gastroplasty with the old mesh and fundus of the stomach being removed (to remove a major source of the appetite stimulating hormone, ghrelin, and also because repeated dissection in this area produces ischaemia and therefore risk of staple line leakage). Hospital length of stay was 6.6 ± 2.6 days. There were no deaths, but 2 patients suffered anastomotic leakage; wound and respiratory complications were common.

## 8. Summary

Conservatively estimated 10% of primary bariatric surgical procedures will eventually fail, and mostly these failures will present as weight recidivism. While clinics for primary bariatric surgery clinics are well established, we are unaware of the existence of a multidisciplinary revision and weight recidivism clinic, as has been established by the provincial Alberta Health Services and has been described above. It is likely that in the future many centres will need to provide resources to manage this complex and growing population of patients.

## Figures and Tables

**Table 1:** Primary weight wise clinic versus bariatric surgical revisional clinic.

A 2012 audit of the primary weight wise clinic versus bariatric surgical revisional clinic, Alberta Health Services		
	Primary bariatric weight wise clinic Primary care referral (*n* = 863)	Weight recidivism & bariatric surgery revisional clinic Primary, secondary, tertiary, and quaternary referral (*n* = 137)
	2012	2012
Age	44.2 ± 11.5 yrs	49.8 ± 10.1 yrs
% ♀	75%	75%
Initial BMI	47.1 ± 7.6	47.0 ± 11.5
Median number of visits pre-op		
Nursing	6	2
Dieticians	7	2
Psychologist	5	3
Exercise specialist	4	0
Internists	3	1
Surgeons	1	2
Prior bariatric surgery %		
LAGB		15%
VBG		79%
Roux-en-Y		5%
Duodenal switch		1%
% of patients receiving surgery	29%	27%
Median number of visits after bariatric procedure		
Nursing	4	2
Dieticians	6	4
Psychologist	3	1
Exercise specialist	2	0
Internists	0	0
Surgeons	4	3
Median amount of time attending clinic after surgery (monthly)	18/12	12
